# Melanonychia with pseudo‐Hutchinson sign may assist in diagnosis of Addison's disease

**DOI:** 10.1111/srt.13441

**Published:** 2023-08-15

**Authors:** Mengyan Zhu, Su Wang, Ping Wang

**Affiliations:** ^1^ Department of Dermatology, Hangzhou Third People's Hospital Affiliated Hangzhou Dermatology Hospital of Zhejiang University School of Medicine Hangzhou China

Dear Editor,

Addison's disease (AD) is a potentially life‐threatening endocrine disorder caused by adrenocortical insufficiency. A deficiency in Adrenocorticotropic Hormone (ACTH) diminishes the negative feedback to the hypothalamic‐pituitary axis, leading the adenohypophysis to secrete elevated levels of plasma ACTH and melanocyte‐stimulating hormone. This hormonal alteration results in skin and mucosal melanin pigmentation.^1^

A woman in her mid‐forties presented with a four‐month history of mucocutaneous pigmentation. The pigmentation, bronzed in appearance, initially appeared on her face and gradually spread across her entire body. It was particularly prominent on the sides of her joints and areas exposed to light. She also developed inhomogeneous grey‐black longitudinal melanonychia in her fingernails and toenails, along with hyperpigmentation in her lips, tongue, and mucosal regions (Figure 1). As the pigmentation evolved, the patient reported a loss of appetite and weight, fatigue, anxiety, and muscular discomfort in the lower limbs. She had an overall good health history, with no oral medication intake for about a year. No individuals with similar symptoms were reported in her immediate family or local community.

**FIGURE 1:**
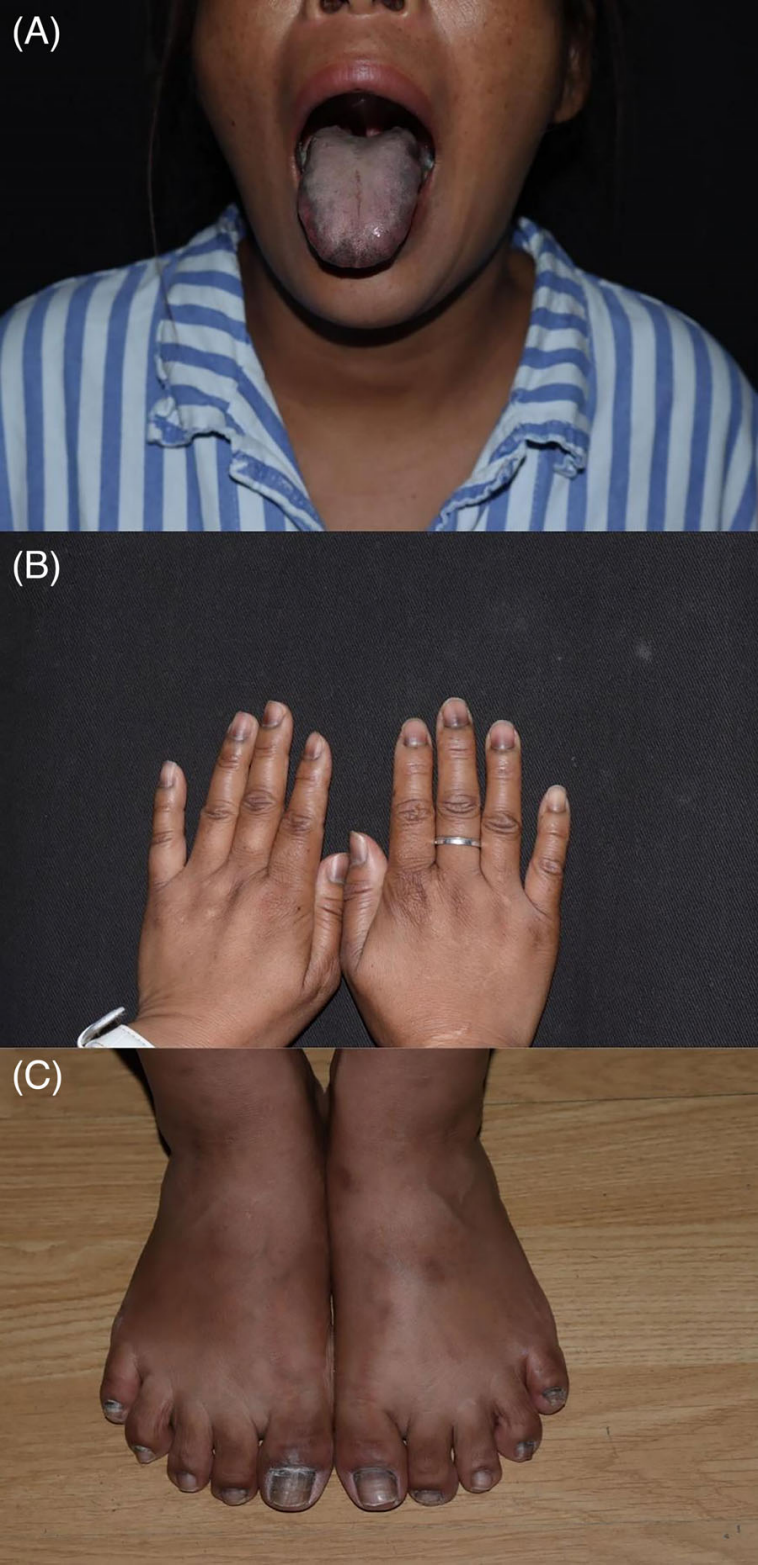
Clinical presentation. (A) Hyperpigmentation in her lips, tongue, and mucosal regions; (B) patient's finger and toenails with grey‐black longitudinal melanonychia.

Upon investigation, we noted an elevated secretion of the patient's prolactin in sex hormones (46.25 ng/mL). Her ACTH level spiked at 8 a.m. (>278 pmol/l), more than twice the upper limit of the standard range, while her cortisol levels were low (2.92 ug/dL at 8 a.m.; 1.18 ug/dL at 4 p.m.;1.79 ug/dL at midnight). The patient's antinuclear antibody, rheumatoid factor, serum electrolyte, and thyroid function all appeared normal. Abdominal enhanced computed tomography scans revealed a left adrenal tumor of approximately 1.2 × 0.6 cm, suspected to be an adenoma. Hypophysis‐enhanced magnetic resonance imaging did not reveal any pituitary abnormalities.

Given the irregular hormone levels, we diagnosed the patient with AD. Following a course of oral hormone therapy, the patient's skin and mucosal pigmentation significantly improved.

In this case, the patient exhibited an inhomogeneous longitudinal brown pigment band under the nails (subungual) and around the nails (periungual). However, a dermatoscopic examination of the nail fold presented normal results, indicating a negative Hutchinson's sign (Figure 2). Hutchinson's sign, characterized by the extension of brown‐black pigment from the nail bed, matrix, and nail plate into the adjacent cuticle and proximal and lateral nail folds, is a diagnostic feature of subungual melanoma.^2^ In contrast, hyperpigmentation of the nail bed may be visible through the “transparent” nail folds, thus mimicking Hutchinson's sign. While melanonychia in AD has been reported in several studies, no identification of Hutchinson's sign in association with AD has been made.^3^, ^4^ The normal appearance of the nail folds in this patient suggests a pseudo‐Hutchinson sign, a feature that can be associated with AD.

**FIGURE 2:**
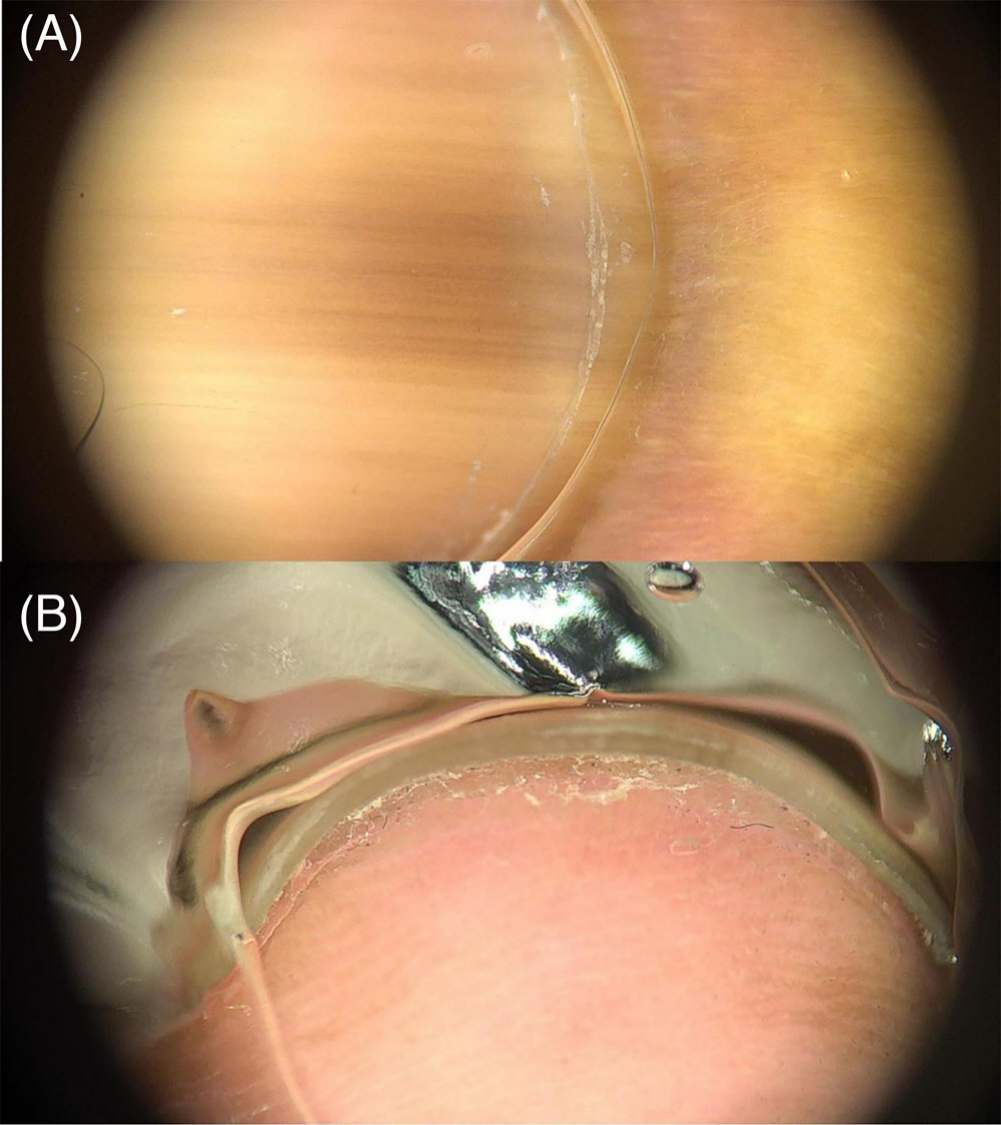
Melanonychia with pseudo‐Hutchinson sign under dermoscopy. (A) Subungual inhomogeneous linear band of brown pigmention and periungual pigmentation. (B) Normal nail fold.

Therefore, AD should be considered in cases of mucocutaneous pigmentation in conjunction with nail discoloration. Inhomogeneous longitudinal melanonychia accompanied by a pseudo‐Hutchinson sign may serve as an indicator of nail changes in AD, assisting in its diagnosis.

## CONFLICT OF INTEREST STATEMENT

The authors declare that there is no conflict of interest that could be perceived as prejudicing the impartiality of the research reported.

## FUNDING INFORMATION

Zhejiang Natural Fund Project, Grant Number: LBZ22H160001

## Data Availability

Data sharing is not applicable to this article as no datasets were generated or analyzed during the current study.
